# EMERGING PALLIATIVE CARE INTERVENTIONS FOR HOMELESS ADULTS IN 10 US CITIES

**DOI:** 10.1093/geroni/igad104.1191

**Published:** 2023-12-21

**Authors:** Arden ODonnell

**Affiliations:** Boston University, Boston, Massachusetts, United States

## Abstract

America’s adult homeless population is growing older; its median age is now 50 years of age. Due to harsh living conditions of chronic homelessness, these individuals often experience geriatric conditions in their 50s and 60s and face premature mortality. Yet, little is known about palliative care for this population. A 2017 systematic review of scientific journals revealed only six palliative care intervention studies for homeless adults. Anecdotal evidence, however, suggests community responses are emerging. Focusing on the 10 U.S. cities with the largest adult homeless populations, this exploratory study had two objectives: 1) through a web-based search of the “grey literature,” the identification of organizations developing or adapting programs to serve the palliative care needs of homeless adults; and 2) through in-depth zoom interviews with directors, gaining an understanding of the programs’ core aspects as well as their successes and challenges in development, implementation, and sustainability. The 18 interviewed agencies fell into two distinct service categories, healthcare/palliative organizations and homelessness/housing organizations. Identified modifications included targeted advanced care planning (ACP) groups, the development of an ACP repository for unhoused persons, rapid re-housing for terminally ill patients, harm reduction strategies, and hospice options in shelters. Common themes were the challenges of working across multiple systems and the lack of dedicated resources and funding for initiatives to support the population’s multiple and complex needs. The discussion highlights the need for greater organizational resources and flexibility in both the healthcare and homeless systems to promote value-based-goal concordant care for homeless adults.

